# Choosing dyes for cw-STED nanoscopy using self-assembled nanorulers

**DOI:** 10.1039/c4cp00127c

**Published:** 2014-03-06

**Authors:** Susanne Beater, Phil Holzmeister, Enrico Pibiri, Birka Lalkens, Philip Tinnefeld

**Affiliations:** a NanoBioSciences Group , Institute for Physical and Theoretical Chemistry , TU Braunschweig , Hans-Sommer-Str. 10 , 38106 Braunschweig , Germany . Email: b.lalkens@tu-bs.de ; Email: p.tinnefeld@tu-bs.de

## Abstract

Superresolution microscopy is currently revolutionizing optical imaging. A key factor for getting images of highest quality is – besides a well-performing imaging system – the labeling of the sample. We compared the fluorescent dyes Abberior Star 488, Alexa 488, Chromeo 488 and Oregon Green 488 for use in continuous wave (cw-)STED microscopy in aqueous buffer and in a durable polymer matrix. To optimize comparability, we designed DNA origami standards labeled with the fluorescent dyes including a bead-like DNA origami with dyes focused on one spot and a DNA origami with two marks at a designed distance of ∼100 nm. Our data show that all dyes are well suited for cw-STED microscopy but that the optimal dye depends on the embedding medium. The precise comparison enabled by DNA origami nanorulers indicates that these structures have matured from the proof-of-concept to easily applicable tools in fluorescence microscopy.

## Abstract 2

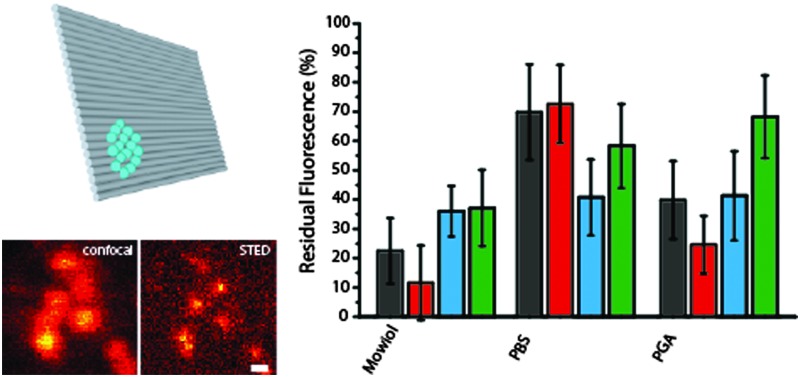
DNA origami used as a platform to study the bleaching behavior of four commonly used fluorophores in cw-STED nanoscopy.

## Background

The theoretical and later on also experimental breaking of the diffraction barrier in light microscopy almost 20 years ago^1,2^ depicts a turning point for any science using light microscopy, because details previously inaccessible by light microscopy became resolvable.

In the last few years, substantial technical developments took place,^3^ making superresolution microscopy exercisable to a considerably larger target audience by not being dependent on specialized optics labs. Especially, commercially available superresolution systems, which are fully and automatically aligned and easy to use, pave the way for superresolution microscopy to become a standard technique in biological and medical research.

Nowadays, there are several, in many regards, complementary techniques to break the diffraction barrier.^4^ Here we focus on Stimulated Emission Depletion (STED) microscopy, which has already shown to improve resolution by almost two orders of magnitude^5^ and is compatible with 3D live imaging of cells, tissue and even transgenic animals.^6–8^ In STED microscopy, the diffraction limited excitation spot is overlayed with a doughnut-shaped spot of a longer wavelength, featuring a local minimum of zero intensity at the very center. The doughnut-shaped light immediately de-excites fluorophores back into the electronic ground state S_0_, thereby effectively switching them off. In the very center, where the de-excitation intensity is zero, however, the fluorophores are not switched off, giving rise to a fluorescence signal which is constrained to a subdiffraction-sized spot. By scanning this spot through the sample, a superresolution image is obtained.

For getting first class superresolution images, in addition to a well-adjusted microscope, the quality and composition of the sample is of crucial importance. Because the signal is collected from a smaller area than that in conventional microscopy, the labeling of the sample has an extensive influence on the obtainable image.^9^ Due to the higher requirements of the fluorophore, care has to be taken to collect as many photons as possible before photobleaching. Besides optimizing conditions like scanning speed, the excitation and STED intensities and the filter settings, this includes the choice of the best fluorophore for the applied settings.

However, systematic studies on the photophysical behavior of fluorophores under STED conditions, although crucial for the successful implementation of STED microscopy, are to our knowledge missing. One possible reason for this shortcoming might be the lack of an adequate test specimen. For a precise comparison, such a sample has to fulfill several requirements: a well-defined number of fluorophores have to be located in a subresolution-sized area, and be exposed to the embedding medium.^10,11^ Since in STED, unlike in PhotoActivated Localization Mikroscopy (PALM), Stochastic Optical Reconstruction Microscopy (STORM) and related superresolution techniques,^12–16^ mostly more than one fluorophore is excited simultaneously, single-molecule bleaching experiments do not reflect the experimental conditions normally applied. Fluorescent beads like YellowGreen-beads are a useful tool for roughly checking if the microscope is properly adjusted, but the signal intensities are much higher than in most real-world samples. Furthermore, they are limited concerning the choice of dyes and control over the exact number of dyes, and the fluorophores are not exposed to the embedding medium, which has a substantial influence on the behavior of the fluorophore. Using antibody-stained samples like actin or single antibodies immobilized on a surface, as is now commonly done in many labs^17^ due to the absence of a better alternative, does not allow a fair comparison either, because the number of fluorophores attached to the antibody is not precisely defined and varies in most cases between 3 and 8.

Therefore, we decided to use DNA origami^18^ as a platform for studying the properties of different fluorophores under cw-STED conditions. The idea behind the assembly of a DNA origami is that a long strand of DNA, called a scaffold, can be forced into basically any three-dimensional shape by the addition of short (app. 50 nucleotides long) complementary single-stranded oligonucleotides. These structures can be used as molecular breadboards where small molecules like fluorophores or biotins can be precisely arranged.

This approach has already demonstrated its potential in the construction of two- and three-dimensional superresolution nanorulers.^19,20^ Such DNA origami nanorulers have also been used to validate approaches for counting molecules and as comparison samples for biological structures.^21^

Here, we use a modified DNA origami design for the comparison of four fluorescent dyes which are commonly used for continuous-wave (cw)-STED microscopy using an excitation wavelength of 491 nm and a STED wavelength of 592 nm. This is probably the most commonly used combination for cw-STED at the moment, because it allows for live cell imaging using common and bright fluorescent proteins like Yellow Fluorescent Protein YFP. Because this wavelength combination is also commercially available, the performance of these dyes is of particular importance for biological and medical labs. To screen conditions for cw-STED microscopy, we designed a bead-like DNA origami which offers a practically free choice of dyes and a defined number of fluorophore molecules per mark. The bead DNA origami is based on a rectangular DNA origami structure,^18,22,23^ where fluorescent molecules can be bound within a small area of about 300 nm^2^. A different DNA origami was then used to test the results obtained with the bead like DNA origami: a nanoruler based on a twelve-helix-bundle construct (12HB) as published in 2012 in Science by Derr *et al.* ^24^ was labeled with 2× 17 dyes at a distance of 100 nm. This nanoruler allows the simple illustration of the resolving power of the microscope.

## Experimental section

### Microscope

STED measurements were performed on a home-built STED microscope. For excitation, a cw laser diode (Cobolt, http://www.cobolt.se) at a wavelength of 491 nm was focused using a 1.4-NA objective lens (UPSLAPO100xO, oil immersion, Olympus, ; http://www.olympus.de). The fluorescence was collected using the same lens and separated from the excitation beam using a custom-made dichroic mirror (zq 491 RDC, AHF, ; http://www.ahf.de/). It was filtered using a 535/70 bandpass filter (ET Bandpass 535/70, AHF ; http://www.ahf.de/) and detected using an avalanche photo diode (Perkin Elmer, ; http://www.perkinelmer.de/) with a multimode optical fiber (diameter 62.5 μm) serving as a confocal pinhole.

Stimulated emission was accomplished using a cw solid state laser (MPBC, http://www.mpbc.ca/) at 592 nm. Excitation beam and STED beam were overlayed using a custom made dichroic mirror (z 590 sprdc, AHF, ; http://www.ahf.de/). A doughnut-shaped laser beam was produced using a helical phase ramp (RPC Photonics, ; http://www.rpcphotonics.com/).

Sample scanning was realized using a scan head consisting of two galvanometric mirrors (Yanus IV Scan Head, TILL Photonics, http://www.fei.com/). Control over both the hardware and detection was performed using the software Imspector.^25^

### Sample preparation

Unmodified as well as biotinylated DNA oligonucleotides in HPLC purification grade were purchased from MWG Eurofins (http://www.eurofinsgenomics.eu/). Fluorescently labeled oligonucleotides in the PAGE purification grade were purchased from IBA (; http://www.iba-lifesciences.com). Prolong Gold Antifade® (PGA) was purchased from Invitrogen (; http://www.lifetechnologies.com). All other chemicals were purchased from Sigma Aldrich (; http://www.sigmaaldrich.com).

All DNA origamis were functionalized with biotin anchors on the bottom side of the structure. The bead DNA origami carries 6 biotin modifications, the 12HB 5 biotins.

### Bead DNA origami

Biotinylated bead DNA origamis were assembled with a 10 fold excess of the staple strands with respect to the scaffold strand (10 nM, 7249 nt long) in Tris-acetate-EDTA (TAE) buffer (40 mM Tris, 2 mM EDTA, 12.5 mM acetate) containing 11 mM MgCl_2_ using a thermal ramp from 95 °C to 4 °C over 2 hours. Excessive staple strands were removed by filtration using an Amicon filter system (100k, 8 minutes, 12k rcf). The sample was recovered in 3 minutes at 3k rcf.

#### 12HB

Biotinylated 12HBs were assembled with a 10 fold excess of staple strands with respect to the scaffold strand (10 nM, 8064 nt long) in TAE buffer containing 16 mM MgCl_2_ using a fast folding method (2 hours at 47 °C).^26^ Excessive staple strands were removed by filtering with an Amicon filter system (100k, 8 minutes, 12k rcf). The sample was recovered in 3 minutes at 3k rcf.

#### External labeling

External labeling was accomplished by mixing the unmodified DNA origamis (approx. 25 μL) with 70 μL of a buffer (TAE, 50 mM NaCl, 12 mM MgCl_2_) and incubated for 2 hours at 37 °C with the complementary oligonucleotide (100 nM; sequence: dye-5′-TTT GTG ATG TAG GTG GTA GAG GAA). Samples were purified with an Amicon filter system by filtering three times (10 minutes, 12k rcf) with TAE + 11 mM MgCl_2_. The samples were recovered in 3 minutes at 3k rcf.

#### Surface preparation

All measurements were performed on bovine serum albumin (BSA)/biotin–neutravidin surfaces (incubation of 1 mg mL^–1^ BSA/biotin for 15 minutes, washing with phosphate buffered saline (PBS), incubation of 0.25 mg mL^–1^ neutravidin for 15 minutes, washing with PBS containing 100 mM MgCl_2_). Measurements in buffer (*e.g.* PBS + 100 mM MgCl_2_) were performed in home-made flow chambers featuring a #1.5 coverslip and a volume of approximately 25 μL sealed with twinsil® (Picodent, ; http://www.picodent.de/) after incubation and washing of the sample.

#### Embedding

For the embedding in PGA, samples were immobilized on #1.5 coverslips and embedded according to the manufacturer's protocol (*e.g.* surfaces were dried before application of the embedding media). The embedded samples were dried overnight at room temperature and sealed with twinsil®. Samples in mowiol were prepared the same way as in PGA.

#### Atomic force microscopy (AFM) imaging

All measurements were performed on a NanoWizard® 3 ultra AFM (JPK Instruments AG, http://www.jpk.com) in TAE. A freshly cleaved mica surface (Quality V1, Plano GmbH, ; http://www.plano-em.de/) was incubated with a 10 mM NiCl_2_ solution for 2 minutes, rinsed with milliQ water and dried in air. The bead DNA origami sample (stock was diluted 1/10 in TAE buffer) was added to the surface and incubated for 2 minutes. The surface was then washed five times with TAE buffer to remove unbound samples. The images were recorded in the tapping mode using high dense carbon ultra-short cantilevers (330 kHz, and 0.3 N m^–1^, Nanoworld, ; http://www.nanoworld.com/). The JPK Data Processing Software was used for analyzing the images.

### Instrumental parameters

Confocal bleaching was minimized by adjusting the parameters of the confocal measurement to less than 5% confocal bleaching per scan.

Parameters for the confocal measurements were 0.05 ms per pixel scanning speed, 50 nm pixel size for all but Chromeo 488 in Mowiol, Oregon Green 488 in PBS and Oregon Green 488 in Mowiol (100 nm pixel in size).

The excitation power was adjusted to 1000 ± 150 photon counts per integrated spot. The excitation power was 3 μW for Alexa 488 in PBS, Abberior Star 488 in PBS; 6 μW for Alexa 488 in PGA, Chromeo 488 in PBS and PGA and Oregon Green 488 in PGA; 9 μW for Alexa 488 in Mowiol, Abberior Star 488 in Mowiol and PGA and Oregon Green 488 in PBS; 12 μW for Chromeo 488 in Mowiol and Oregon Green 488 in Mowiol; laser power was measured in the aperture of the objective lens.

All STED measurements were performed under the same conditions to ensure comparability. The scanning speed was 0.05 ms per pixel; the pixel size was 25 nm. The STED laser was turned to full power for all experiments. Full STED power measured in the aperture was 370 mW.

#### Simulations

Monte Carlo simulations were performed using custom-made Labview software for two two-dimensional Gaussian functions with a given FWHM at a given distance. To ensure comparability, the simulation parameters were chosen according to the real measurement parameters: FWHM was set to 180 nm and 90 nm for the confocal and STED simulations, respectively. The distance was set to 100 nm, the pixel size to 25 nm and the number of photons was 1000 photons per spot. The simulations underlie shot-noise but no background.

## Results and discussion

**Fig. 1:**
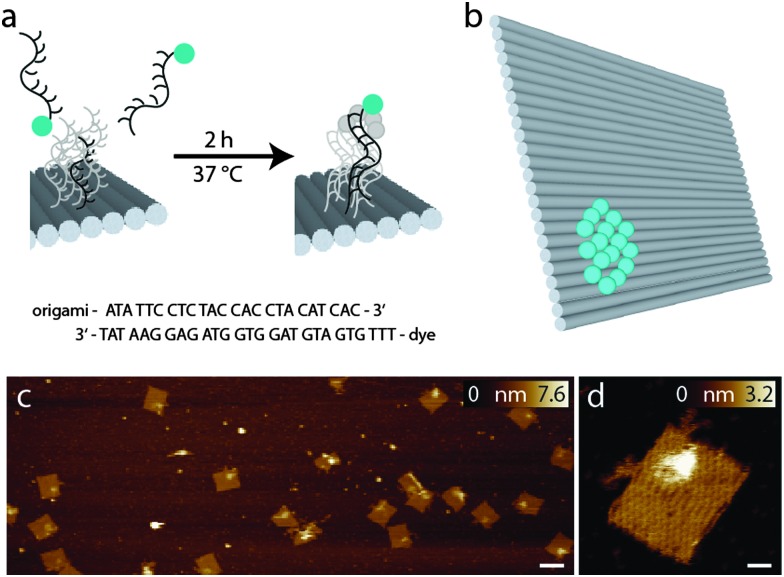
(a) External labeling principle; (b) scheme of the bead DNA origami with the ideal number of 16 fluorophores corresponding to 100% labeling yield; (c) AFM image of the bead DNA origamis, the scale bar is 100 nm; (d) enlarged view of a bead DNA origami, the area of the extended surface docking strands with complementary dye labeled strands can be clearly identified, the scale bar is 10 nm.

To compare the performance of different fluorophores for cw-STED microscopy, we designed a so-called bead DNA origami, which is based on a rectangular structure originally described by Paul Rothemund^18^ in a slightly modified way that minimizes distortion.^22,23^ In a small area of about 300 nm^2^, the 3′ ends of the staple strands facing the top of the DNA origami were extended by a 24 nucleotide sequence, which serves as an anchor for binding complementary fluorophore-modified oligonucleotides, as can be seen in Fig. 1a. This so-called external labeling has several advantages: only one modified staple strand for each fluorophore has to be synthesized, which minimizes costs and effort. Furthermore, the same DNA origami structure can be used for the labeling with different fluorophores, excluding unwanted side effects like variations in folding or labeling efficiencies, and therefore, making different samples comparable.

The advantage of the bead DNA origami is the predetermination of the number of fluorophores and the exposition of the fluorophores to the solvent. Unlike in conventional fluorescent beads, the fluorophores are directly accessible to different embedding media, therefore also allowing for the comparison of their influence on the photophysical properties. In contrast to fluorophore-labeled antibodies, which are so far the method of choice for comparing different dyes,^17^ the number of fluorophores bound is well defined with the bead DNA origamis, and can be adapted to match experimental conditions.

Fig. 1c shows representative AFM images of the bead DNA origamis labeled with fluorophores and electrostatically immobilized on mica surfaces. As can be seen in the overview (left), most of the rectangular structures are well folded; only few truncations and deflections are visible. The bound fluorophores can be seen as a light-colored area on top of the DNA origami in one corner. Since the binding to the surface in this case is based on electrostatic interaction, the orientation of the DNA origami is random, resulting in a stochastic distribution of DNA origamis facing downwards and DNA origamis with the fluorophores facing upwards. However, Fig. 1c shows a preferred orientation of the DNA origami structures with labeled fluorophores on top.

For specific binding on the surface opposite to the fluorophore-carrying surface, the lower side of the DNA origamis was labeled with biotin, which binds to a BSA–neutravidin covered surface and therefore gives rise to a predominant direction with the fluorophores facing upwards into solution. In Fig. 1d, a detailed view of a single DNA origami is shown. In addition to the helical structures establishing the rectangular DNA origami, a single-stranded unstructured loop is visible on the upper left edge, which is a leftover of the scaffold strand not used for the rectangle.

Next, we scrutinized the bead DNA origamis by STED microscopy.

**Fig. 2:**
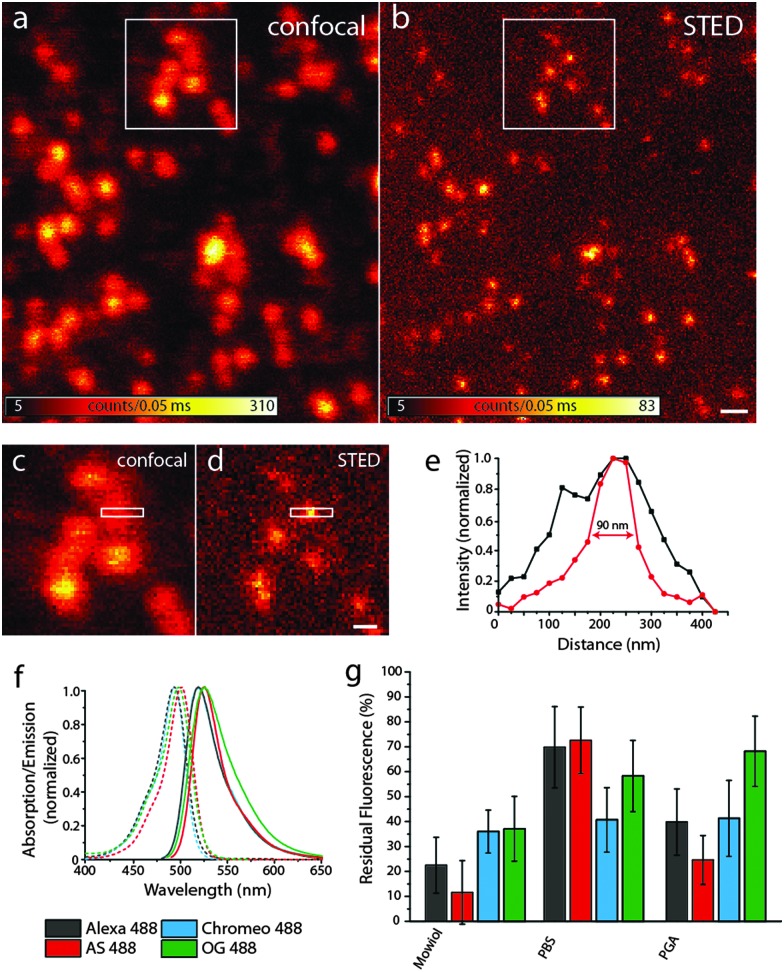
(a) and (b) Confocal and STED images of Alexa 488-labeled bead DNA origamis bound to a BSA–neutravidin-surface, the scale bar is 400 nm; (c) and (d) zoom of the areas indicated by the white rectangles in (a) and (b), scale bar 200 nm; (e) intensity profile of the area indicate by the white rectangles in (c) and (d), black: confocal profile, red: STED profile; (f) spectra of the dyes used; (g) residual fluorescence after one STED scan for the four dyes in different media.

Fig. 2a and b show representative confocal and STED images, respectively, of bead DNA origamis labeled with Alexa 488. The STED image (b) shows that the individual beads are well resolved, which is not always the case in the confocal image (a). The full-width-half-maximum (FWHM) in the STED images is measured to be around 90 nm, as shown in Fig. 2e. Adding up three rows of pixels, indicated by the white rectangle in the zoomed-in regions (Fig. 2c and d), results in a clearly narrower profile for the STED image that demonstrates resolution beyond the diffraction limit. The obtained resolution enhancement resembles both the theoretical expectations for these settings as well as experiments using fluorescent beads.

To compare the bleaching behavior of these dyes under STED conditions, we took serial images of the respective fluorophore-labeled DNA origamis: after a confocal scan, a STED image of the same region with a maximal STED power was recorded, followed by a second confocal scan.

The residual fluorescence intensity in the second confocal image was compared to the first, pre-STED scan. Therefore, spots were detected in the first confocal image, the brightness was determined by spot integrated statistics and compared to the relative spot integrated brightness at the same position in the last confocal scan. Care was taken so that no bleaching could occur in confocal imaging, so all the intensity loss was due to the STED scan.

In addition to comparing different fluorophores, we also used different embedding media which are common in (superresolution) fluorescence microscopy: PBS (phosphate-buffered saline) is a water-based buffer often used for monitoring biological processes, whereas Mowiol and PGA are polymers which allow for the long-term storage of the samples. Oxygen-depleted buffers lead to pronounced blinking; therefore, we did not pursue this approach any further.

The intensity of the STED laser was set to the maximum power to achieve the highest resolution obtainable with these settings. The excitation intensity was adapted so that the detected fluorescence intensity in the STED images was the same for all samples. Apparently, the excitation intensity is a key factor for bleaching in STED imaging and therefore has to be tightly controlled when comparing different fluorophores and the dependence of the embedding medium.

We compared four different dyes often used in STED microscopy, namely Alexa 488, Abberior Star 488, Chromeo 488 and Oregon Green 488.^27^ As shown in Fig. 2f, the absorption and emission spectra of these dyes are very similar, which leads to the assumption that the resolution increase for a given STED-power is similar for all dyes. Other dyes in the same spectral region like fluorescein were discarded, since their photostability under STED conditions was too low to obtain decent STED images.

As can be seen in Fig. 2g, there are considerable differences in the bleaching behavior: Chromeo 488 only shows moderate bleaching resistance with a residual fluorescence of only about 30% in all embedding media. Oregon Green 488 is a good choice for STED imaging in PGA, whereas in Mowiol and PBS, there are better alternatives. Both Alexa 488 and Abberior Star 488 perform best in PBS with a residual fluorescence intensity of more than 70%, which would also allow acquisition of more than one consecutive STED image.

As discussed before, bleaching strongly scales with the excitation intensity in STED imaging. Generally, it has to be adjusted to obtain a reasonable signal-to-noise ratio in the STED image and therefore strongly depends on the sample and especially the fluorophore density per pixel. In this example, about 7 fluorophores were located in a circular area with a diameter of 20 nm, as determined by comparing the brightness of single fluorophores immobilized on the surface. This number mimics a sparsely labeled immunostained sample. For samples more densely labeled, as is the case for most applications, the number of fluorophores might be higher. For rather dim samples, however, we conclude that if there are no other restrictions on the choice of fluorophore (such as membrane permeability or labeling strategy) the optimal fluorophore depends on the embedding medium: the photostability of Alexa 488 and Abberior Star 488 is clearly the best in PBS whereas Oregon Green 488 bleaches least in PGA and Chromeo 488 is hardly affected by the medium. Therefore, for the imaging of a biological specimen in buffer, Alexa 488 and Abberior Star 488 should be preferred. Oregon Green 488 is less photostable in PBS but as it can penetrate the cell membrane, it should definitely be considered for live cell experiments. Also for the use in embedded samples, our data recommend the use of Oregon Green 488 along with PGA as a medium. Chromeo 488 shows a moderate photostability in all media tested in this study. This consistency might be useful for comparative studies. Additionally, all dyes tested here were least photostable in Mowiol. Therefore, our data recommend the use of *e.g.* PGA for the embedding of fluorescently labeled samples.

**Fig. 3:**
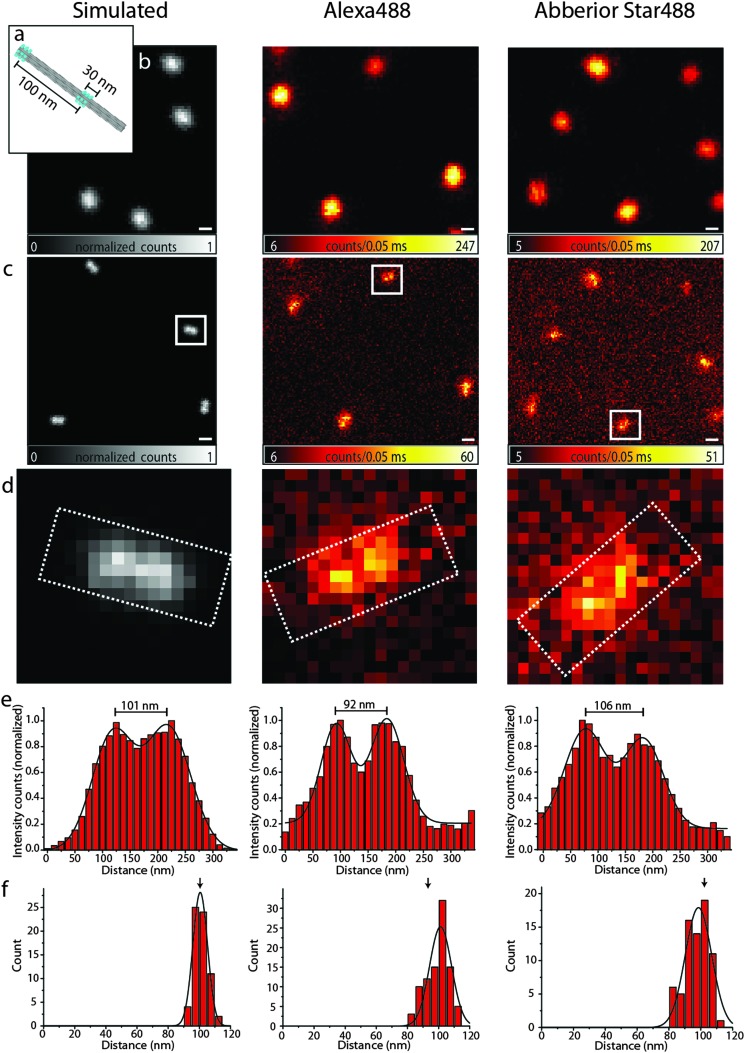
(a) Scheme of the 100 nm DNA origami ruler; (b) confocal images of the 100 nm ruler, left: simulated, middle: Alexa 488, right: Abberior Star 488, scale bar 250 nm; (c) corresponding STED images, the scale bar is 250 nm; (d) zoom of the area in the white rectangles of (c); (e) intensity profiles of the white rectangles in (d); (f) distance distributions of the 100 nm ruler.

Resolution is commonly defined by the ability to distinguish two objects. To substantiate our findings obtained with the bead DNA origamis, we therefore designed a different DNA origami, in the following termed as a nanoruler, where external binding sites of 17 fluorophores in an area of 14 × 30 nm^2^ can be arranged in different distances along a 12 helix bundle (12HB) (see Fig. 3a). With this experimental flexibility, one can consider the optimal distance to check the performance of a system. The most obvious number is obtained with the experimental FWHM and the Rayleigh criterion. The Rayleigh definition of resolution does, however, not consider the limited signal-to-noise and pixelation of the image, which is inherent to experimental data, and a realistic resolvable distance will be larger than that for the FWHM.^28^ To illustrate this issue and to decide which distance of two labeled spots is still resolvable with our settings, we carried out Monte Carlo simulations as shown in Fig. 3, left panels. For a distance of 100 nm in the simulated confocal image (3b, left), the structure cannot be resolved, whereas in the simulated superresolution image with a fixed FWHM of 90 nm, as experimentally determined above, the two features can just be resolved (Fig. 3c and d). The simulated nanorulers consist of two two-dimensional Gaussian functions with a FWHM of 90 nm at a distance of 100 nm.

Therefore, we labeled the 12HB with two external labeling sites with an intermark distance of 100 nm using Alexa 488 and Abberior Star 488, respectively. Fig. 3, middle and right column, shows the confocal and STED images, respectively. As expected, in the confocal images, only a single spot is visible, whereas STED microscopy can resolve the two features with a sufficient signal-to-noise ratio. For the design of DNA origami nanorulers, our data indicate that the optimal distances between two marks should be somewhat larger than those obtained from the Rayleigh criterion to account for pixilation and noise.

The intensity profiles shown in Fig. 3e and the distance histograms (Fig. 3f) were obtained using the software tool CAEOBS.^29^ The software automatically detects the structures, rotates them such that the 12HBs are oriented horizontally and sums vertically over the intensity of all pixels. The resulting one-dimensional intensity histogram is fitted with the sum of two Gaussian functions. The distance between the maxima of the Gaussian functions then provides the distance between the two spots. The distance distribution (Fig. 3f) shows an expected average distance of about 100 nm for each of the three cases (simulated, Alexa 488 and Abberior Star 488). The width of the distributions shown in Fig. 3f is slightly larger for experimental than for simulated data. This is due to different experimental challenges: the naturally given roughness of a BSA–biotin-surface leads to slight distortions of DNA origami structures. Also the 12HBs are not perfectly stiff so the resulting distance between the two marks varies with the degree of distortion.

A different challenge is the automatic analysis.^29^ The rotation required for the distance analysis induces a bias towards smaller distances if the structures are not rotated to a perfectly horizontal orientation. To reduce bleaching and recording times, the pixel size is usually chosen as large as possible which makes the perfect alignment a bit more difficult.

Finally, the STED measurement causes a non-uniform background signal subject to shot-noise, which reduces the homogeneity compared to the background free simulations. In order to minimize this effect, we customized the CAEOBS software for analysis of non-quadratic regions of interest (ROIs) (white rectangles in Fig. 3d), so that we only consider the central region of the nanorulers with a sufficient signal-to-noise ratio.

## Conclusion

In this work, we studied the four preferential dyes Abberior Star 488, Alexa 488, Chromeo 488 and Oregon Green 488 for a popular cw-STED configuration with excitation at 491 nm and 592 nm STED wavelengths. In contrast to *e.g.* fluorescein, all dyes enabled STED imaging with only ∼7 dye molecules on a 20 nm diameter spot that was created on DNA origami structures. The DNA origamis served as unique platforms for placing a defined number of fluorophores on a small, solvent-exposed surface allowing a fair comparison and variation in imaging parameters. Interestingly, depending on the embedding medium, the dyes showed remarkably different photostability. For imaging in PBS, our data recommend the use of Abberior Star 488 or Alexa 488, whereas in PGA, Oregon Green 488 is the most photostable fluorophore. In comparison, Chromeo 488 exhibited relatively environmentally independent average performance in all media.

The results indicate that such DNA origami structures can represent a general platform for testing and quantitatively compare new fluorophores, with regard to different conditions like STED intensities, pulse length, wavelength, embedding media *etc.* and a combination thereof. Such a reproducible platform simplifies the design of new experiments in (cw-)STED or other superresolution microscopy.

## References

[cit1] Hell S. W., Wichmann J. (1994). Opt. Lett..

[cit2] Klar T. A., Hell S. W. (1999). Opt. Lett..

[cit3] Hell S. W. (2007). Science.

[cit4] Hell S. W. (2009). Nat. Methods.

[cit5] Rittweger E., Han K. Y., Irvine S. E., Eggeling C., Hell S. W. (2009). Nat. Photonics.

[cit6] Westphal V., Rizzoli S. O., Lauterbach M. A., Kamin D., Jahn R., Hell S. W. (2008). Science.

[cit7] Hein B., Willig K. I., Hell S. W. (2008). Proc. Natl. Acad. Sci. U. S. A..

[cit8] Berning S., Willig K. I., Steffens H., Dibaj P., Hell S. W. (2012). Science.

[cit9] Wurm C., Neumann D., Schmidt R., Egner A. and Jakobs S., in Live Cell Imaging, ed. D. B. Papkovsky, Humana Press, 2010, vol. 591, pp. 185–199.

[cit10] Cordes T., Strackharn M., Stahl S. W., Summerer W., Steinhauer C., Forthmann C., Puchner E. M., Vogelsang J., Gaub H. E., Tinnefeld P. (2010). Nano Lett..

[cit11] Yabiku Y., Kubo S., Nakagawa M., Vacha M., Habuchi S. (2013). AIP Adv..

[cit12] Rust M. J., Bates M., Zhuang X. (2006). Nat. Methods.

[cit13] Betzig E., Patterson G. H., Sougrat R., Lindwasser O. W., Olenych S., Bonifacino J. S., Davidson M. W., Lippincott-Schwartz J., Hess H. F. (2006). Science.

[cit14] Hess S. T., Girirajan T. P., Mason M. D. (2006). Biophys. J..

[cit15] Heilemann M., van de Linde S., Schuttpelz M., Kasper R., Seefeldt B., Mukherjee A., Tinnefeld P., Sauer M. (2008). Angew. Chem., Int. Ed..

[cit16] Steinhauer C., Forthmann C., Vogelsang J., Tinnefeld P. (2008). J. Am. Chem. Soc..

[cit17] Dempsey G. T., Vaughan J. C., Chen K. H., Bates M., Zhuang X. (2011). Nat. Methods.

[cit18] Rothemund P. W. (2006). Nature.

[cit19] Schmied J. J., Forthmann C., Pibiri E., Lalkens B., Nickels P., Liedl T., Tinnefeld P. (2013). Nano Lett..

[cit20] Schmied J. J., Gietl A., Holzmeister P., Forthmann C., Steinhauer C., Dammeyer T., Tinnefeld P. (2012). Nat. Methods.

[cit21] Kurz A., Schmied J. J., Grussmayer K. S., Holzmeister P., Tinnefeld P., Herten D. P. (2013). Small.

[cit22] Woo S., Rothemund P. W. K. (2011). Nat. Chem..

[cit23] Li Z., Wang L., Yan H., Liu Y. (2012). Langmuir.

[cit24] Derr N. D., Goodman B. S., Jungmann R., Leschziner A. E., Shih W. M., Reck-Peterson S. L. (2012). Science.

[cit25] Schonle A., Imspector Image Acquisition & Analysis Software, v0.10, 2006, http://www.imspector.de.

[cit26] Sobczak J. P., Martin T. G., Gerling T., Dietz H. (2012). Science.

[cit27] Moneron G., Medda R., Hein B., Giske A., Westphal V., Hell S. W. (2010). Opt. Express.

[cit28] Beater S., Raab M. and Tinnefeld P., in Quantitative Imaging in Cell Biology, ed. J. Waters and T. Wittmann, Elsevier, 2014, accepted.

[cit29] Schmied J. J. Raab M. Forthmann C. Pibiri E. Wünsch B. Dammeyer T. Tinnefeld P., Nat. Protocols, 2013 , , accepted . 10.1038/nprot.2014.079 24833175

